# USP22 promotes development of lung adenocarcinoma through ubiquitination and immunosuppression

**DOI:** 10.18632/aging.103056

**Published:** 2020-04-15

**Authors:** Bing Han, Yue Sun, Dongdong Yang, Huijuan Zhang, Steven Mo, Xuesong Chen, Hailing Lu, Xueyan Mao, Jing Hu

**Affiliations:** 1 Department of Internal Medical Oncology, Harbin Medical University Cancer Hospital, Harbin, Heilongjiang, China; 2 Department of Surgical Oncology, The Fourth Affiliated Hospital of Harbin Medical University, Harbin, Heilongjiang, China; 3 Nanning Life-Ontology Biotechnology Co., Ltd., Nanning, Guangxi, China; 4 Henan University of Science and Technology, Luoyang, Henan, China

**Keywords:** lung adenocarcinoma, USP22, STAT1, UBC, global regulation network

## Abstract

Ubiquitin-specific protease 22 (USP22) expresses highly in lung adenocarcinoma (LUAD), which are associated with poor overall survival (OS). Microarray processing was performed to determine gene expression profiling, in which it was found that knocking down USP22 resulted in abnormal expression of a large number of genes. Differentially expressed genes (DEGs)-based protein-protein interaction (PPI) network was organized into 9 functional modules. These functional modules participated significantly in protein modification-related biological process and were involved in cancer-related pathways. The network was constructed to describe the global regulation of USP22-TF/pivot-module-pathway. It suggested that knocking down USP22 may up-regulate the expression of UBC to promote the pathways of cell cycle and ubiquitin-mediated proteolysis in the development of LUAD. More than that, knocking down USP22 can up-regulate STAT1 to activate JAK1-STAT1-caspase pathway, and promote apoptosis of tumor cell. Receiver operating characteristic (ROC) curve analysis suggested that E2F3, H2AFX, TFAP2A, PITX1, IRF7, and FOXM1 may be the potential diagnosis biomarkers for LUAD. On the other hand, BRCA1, FOXM1 and TFAP2A may be prognostic biomarkers of LUAD. In conclusion, we constructed a global regulation network to show that USP22 may promote the development of LUAD through ubiquitination and immunosuppression.

## INTRODUCTION

Deubiquitinating enzymes (DUBs) regulate several cellular mechanisms including cell cycle progression, signal transduction, growth and differentiation by catalyzing the deconjugation of ubiquitin-tagged substrates [1–3]. Ubiquitin-specific protease 22 (USP22) is a subunit of DUBs with specific targets of therapeutic importance. During the past decades, much work has been performed to confirm that USP22 is highly expressed in colon cancer, bladder cancer, breast cancer, gastric cancer and other tumors. The abnormal expression of USP22 played an important role in regulating DNA transcription, cell cycle transformation and genomic stability of tumors [4, 5]. USP22 can not only activate some known carcinogens such as BMI-1, c-MYC, but also inhibit the expression of some anti-cancer factors such as TP53 through ubiquitination, thus promoting the proliferation of tumors [6]. Our previous studies have confirmed that the expression of USP22 is generally high in non-small lung cancer (NSCLC), which indicated a worse prognosis [6, 7]. It suggested that USP22 played an oncogene role, which may be a potential therapeutic target in NSCLC. However, it has not been fully clarified how USP22 regulates the exact mechanism and pathway on the progression and metastasis of tumors. Previous studies have confirmed that USP22 can promote the biological process of NSCLC cells by regulating BMI-1/AKT signaling pathway [7]. Moreover, USP22 can also regulate the endocytosis of EGFR by deubiquitination modification, which may lead to the sustained activation of EGFR-dependent signaling pathway. It promotes the resistance of EGFR-TKIs in EGFR-mutant lung adenocarcinoma (LUAD) [8]. However, there is no further study on the molecular mechanism of USP22 in LUAD. At present, little is known about the genes or pathways regulated by USP22. Therefore, we attempted to explore the expression of USP22 in LUAD, as well as the possible regulatory genes and biological processes.

In the present study, we found that USP22 was significantly elevated in LUAD. Knocking down USP22 can result in a series of genetic and functional modules dysfunction. According to the USP22 comprehensive regulation network, we propose that USP22 may promote the development of LUAD through ubiquitination and immunosuppression.

## RESULTS

**Figure 1:**
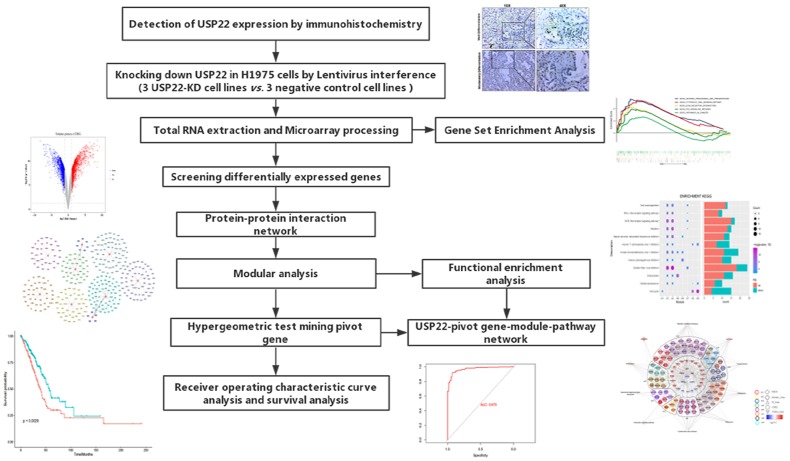
**Flow Chart of this study.**

With the development of molecular transcription research, the gene expression has attracted widespread attention to lung cancer research. According to previous studies, high expression of USP22 in LUAD is usually associated with poor prognosis [7]. The workflow of the present study was presented in Figure 1.

### Knocking down USP22 leads to differential expression of multiple genes in H1975 cell

**Figure 2:**
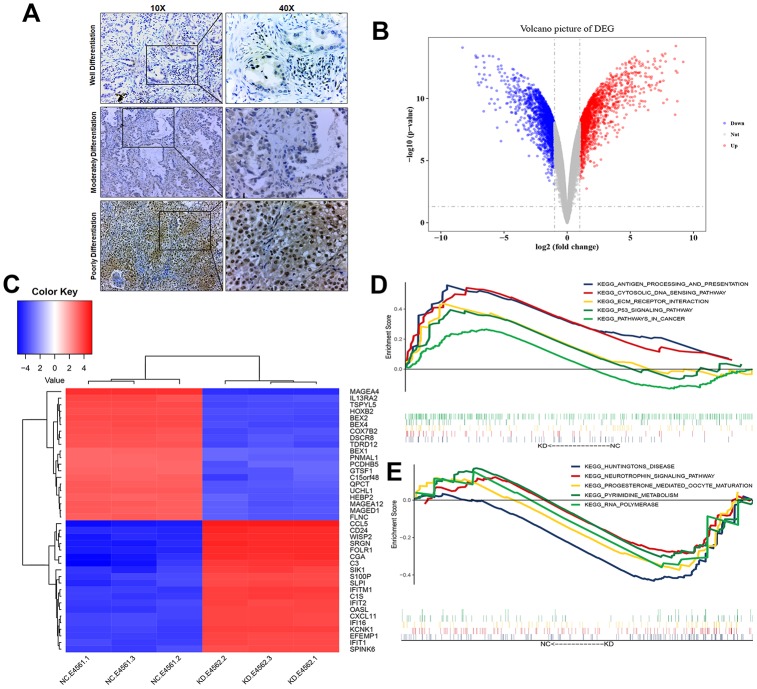
**Transcriptional changes of LUAD H1975 cell line induced by knockdown of USP22.** (**A**) Detection of USP22 by immunohistochemistry. (**B**) Differentially expressed genes in USP22-KD H1975 cells compared to negative control. Red represents up-regulated genes, blue represents down-regulated genes, and gray represents no significantly differentially expressed genes. (**C**) The expression patterns of these 3806 differentially expressed genes can distinguish between USP22-KD H1975 cells and negative control. (**D**) Pathways significantly enriched in negative control H1975 cells. (**E**) Pathways significantly enriched in USP22-KD H1975 cells.

Compared with USP22-negative samples in LUAD, there were poorer differentiation, larger tumor size, and more advanced disease stage in USP22-positive samples (Table 1). This suggested that USP22 may be a key gene to promote the development of LUAD. USP22 was identified to highly express in LUAD through immunohistochemical experiment of the tumor tissues (Figure 2A). Total 3806 differentially expressed genes (DEGs) between USP22-knockdown (USP22-KD) H1975 cells and negative control (NC) (Figure 2B), including 1804 up-regulated genes and 2002 down-regulated genes. It suggested that knocking down USP22 can cause significant changes in RNA transcription of LUAD H1975 cell line. We could accurately distinguish from USP22-KD and NC H1975 cells by the cluster analysis (Figure 2C). Gene set enrichment analysis (GSEA) showed that cancer-related pathways were significantly enriched in USP22-KD H1975 cells (Figure 2D). In addition, these pathways mainly were involved in antigen processing, ECM-receptor interaction, P53 signal pathway and pathways in cancer. Pyrimidine metabolism pathways and RNA metabolic pathway were significantly enriched in NC (Figure 2E). These pathways may be the potential regulatory mechanism of USP22 in LUAD. Therefore, we further explored them at the biological network level.

**Table 1:** Correlation USP22 protein expression level and clinicopathological variables.

**Variables**	**No.**	**USP22**		***P***
		**negative**	**positive**	
Gender				0.088
Male	57	23	34	
Female	53	30	23	
Age(years)				0.846
<60	80	39	41	
≥60	30	14	16	
Differentiation				**0.002**
Well/moderate	44	29	15	
Poor/mucinous	66	24	42	
Tumor size (cm)				**0.039**
≤3	47	28	19	
>3	63	25	38	
Lymphatic Metastasis				0.172
No	51	21	30	
Yes	59	32	27	
AJCC stage				0.013
I~II	73	29	44	
III	37	24	13	
UBC				**<0.001**
- ~ +	46	34	12	
+ + ~ + + +	64	19	45	
STAT1				**<0.001**
- ~ +	45	31	14	
+ + ~ + + +	65	22	43	

### Knocking down USP22 results in dysfunction of multiple modules in H1975 cells

**Figure 3:**
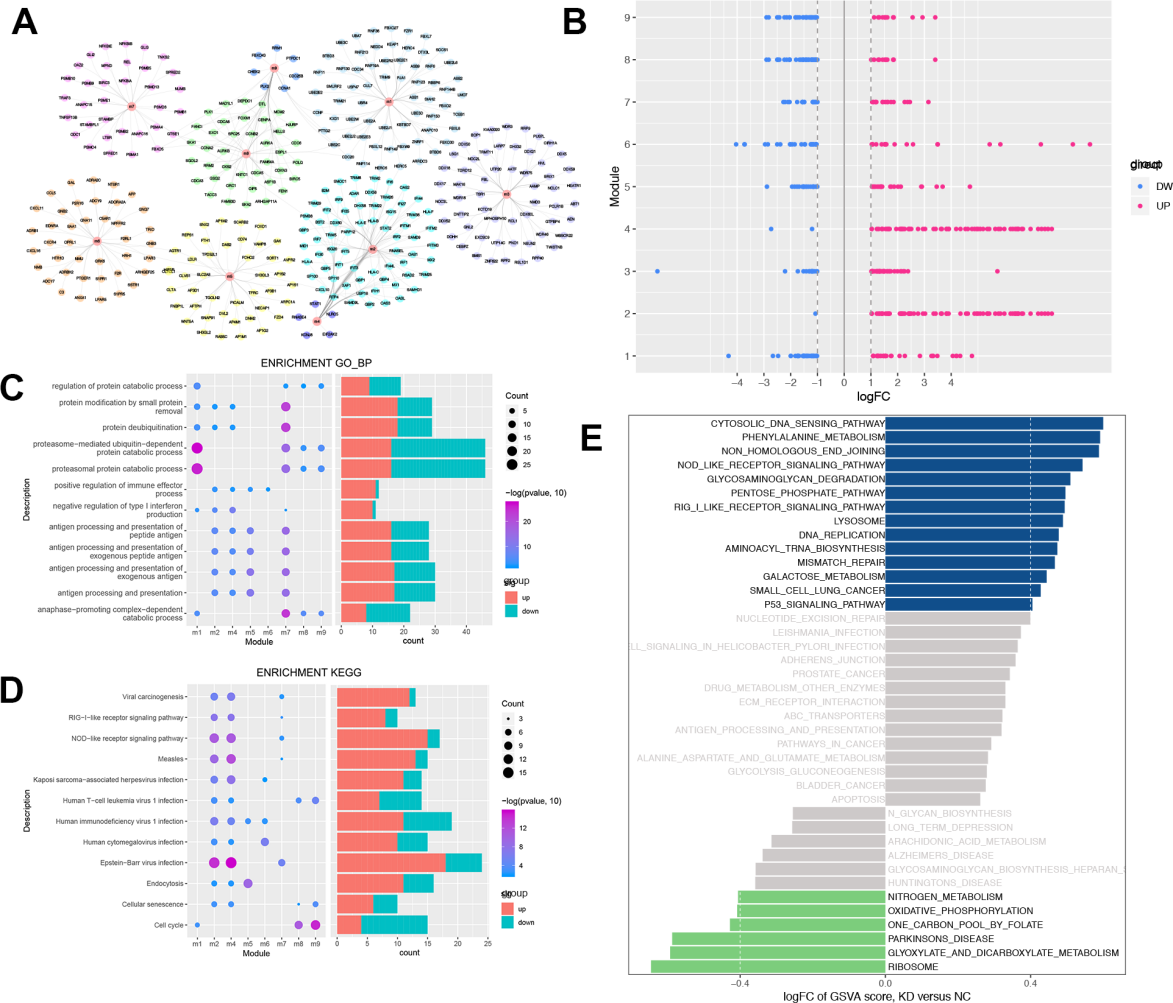
**Modular analysis of protein-protein interaction network and functional enrichment analysis.** (**A**) The modules and the module genes, the colors indicated the different modules. (**B**) Each module contains up-regulated and down-regulated genes. Blue indicates down-regulation of gene expression, while red indicates up-regulation of gene expression. (**C**) Biological process with more than 3 functional modules significantly enriched in. Blue indicates down-regulation of BPs, while red indicates up-regulation of BPs. (**D**) KEGG pathways which more than 2 functional modules enriched in. Blue indicates down-regulation of KEGG pathways, while red indicates up-regulation of KEGG pathways. (**E**) GSEA of KEGG pathway in USP22-KD H1975 cells compared to negative control. LogFC > 0 indicated that the pathway was activated, while logFC < 0 indicated that the pathway was suppressed. Blue indicated the activated pathway was with logFC > 0.4, while green indicated the suppressed pathway was with logFC < -0.4.

Compared to NC, USP22-KD H1975 has changed at both molecular network and pathway levels. Therefore, we mapped DEGS into protein-protein interaction (PPI) network to construct a protein expression disorder network affected by USP22 in LUAD (Supplementary Figure 1). There are 9 functional modules, which are identified from PPI (Figure 3A). They are closely related protein linkers in biological networks. Recognition of modules was helpful for finding more biological subnets in the whole network. All modules contained DEGs of both up-regulated and down-regulated (Figure 3B). Enrichment analysis was carried out for each functional module. Some functional modules were significantly enriched in some biological processes (BPs) (Figure 3C), such as protein modification, protein-deubiquitination, regulation of immune effector process and catabolic process. Meanwhile, some functional modules were enriched in some cancer-related pathways (Figure 3D), such as viral carcinogenesis, NOD-like receptor signaling pathway, virus infection, endocytosis, cellular senescence, and cell cycle. Gene Set Variation Analysis (GSVA) indicated that knocking down USP22 could result in dysfunction in multiple pathways, which involved in cytoplasmic DNA sensing pathway, RIG-I-like receptor signaling pathway lysosomes, DNA replication, mismatch repair, p53 signaling pathway, nitrogen metabolism, oxidative phosphorylation, and ribosome pathway (Figure 3E).

### USP22 may regulate modules through multiple pivot genes and transcription factors (TFs)

**Figure 4:**
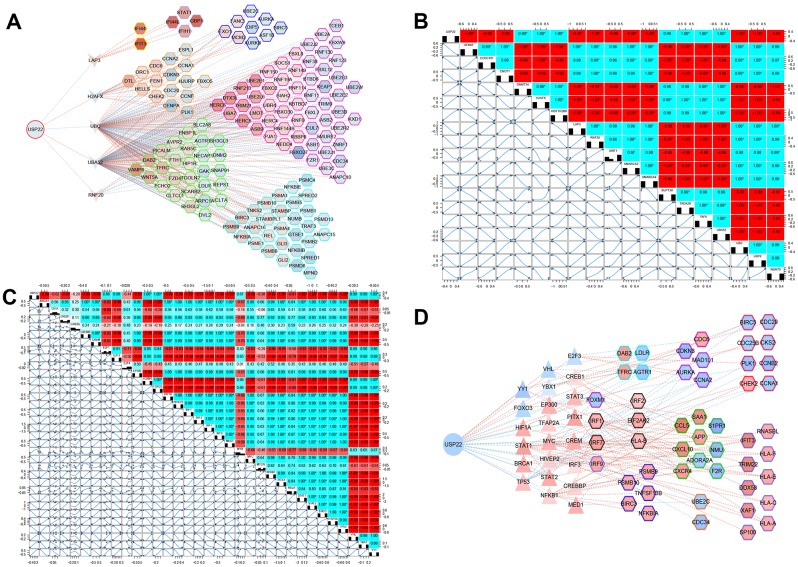
**Potential key pivot and transcriptional factors of LUAD regulated by USP22.** (**A**) USP22 regulates multiple modules through pivot genes. (**B**) The transcription factors correlated with USP22. (**C**) The transcription factors correlated with its target genes. (**D**) USP22 regulates multiple modules through transcription factors.

The results of hypergeometric test showed that LAP3, H2AFX, UBC, UBA52, and RNF20 may be potential pivot genes to regulate modules (Figure 4A). In addition, there was a significant correlation between 5 TFs and their target genes (Figure 4B). It suggested that USP22 might regulate its downstream modules and pathways, not only by directly down regulating the expression of LAP3, UBC and RNF20, but also by up regulating the expression of H2AFX and UBA52. Thus, these regulations might further affect the development of LUAD. In addition, we found that USP22 was significantly related to 33 TFs. Among them, 26 TFs may play a potential regulator role in these module genes according to hypergeometric test (Figure. 4C). Therefore, we inferred that USP22 may regulate the downstream modules and pathway networks by influencing these 26 transcription factors (Figure. 4D). These pivot genes involved in pathways ubiquitin-mediated proteolysis, lysosome, virus infection and cell cycle.

### Comprehensive landscape of USP22-KD regulation in LUAD

**Figure 5:**
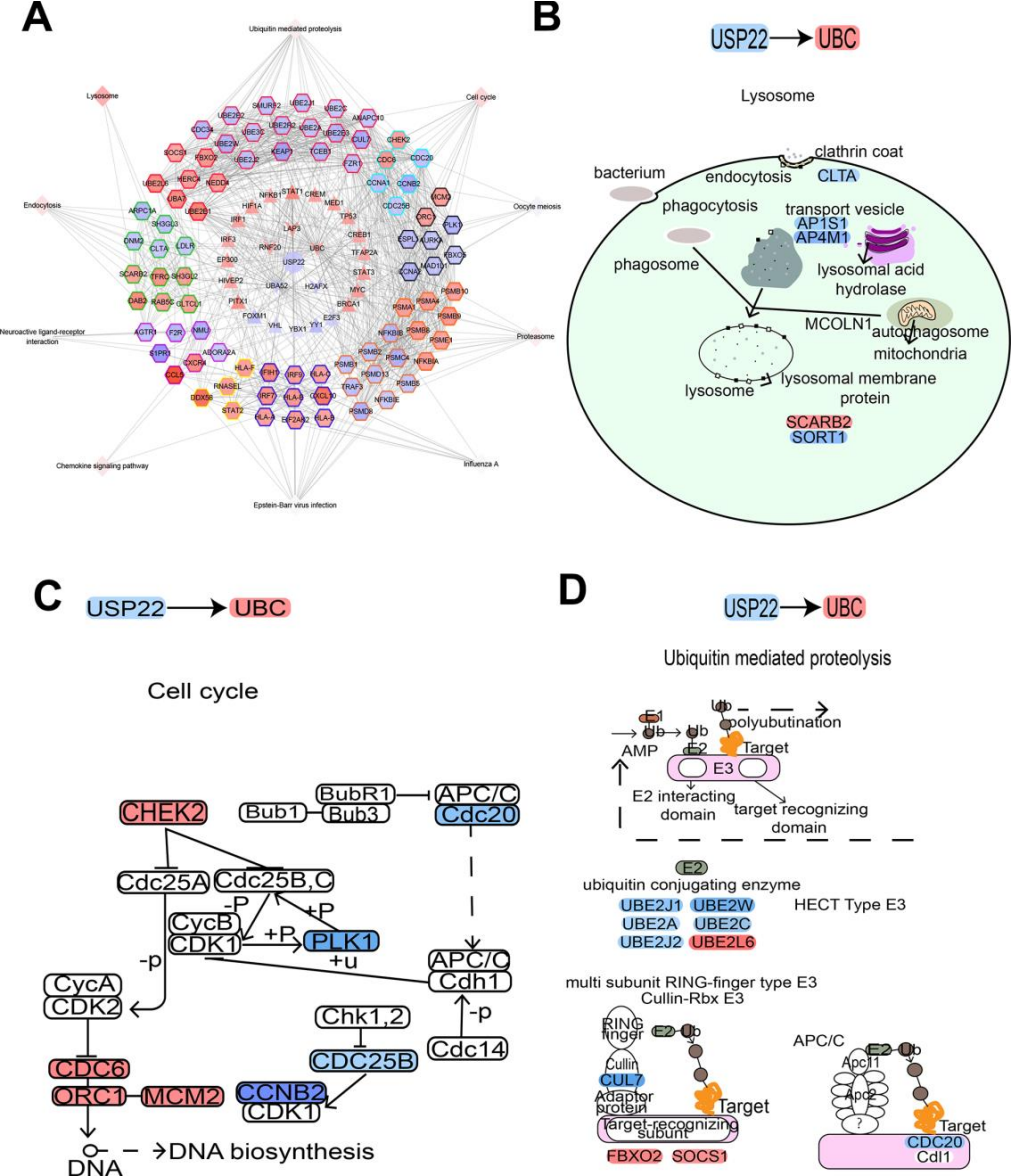
**USP22-pivot gene-module-pathway network suggesting USP22 could affect multiple pathways by up-regulating UBC.** (**A**) USP22 regulates the modules by regulating pivot genes in the development of LUAD. (**B**) Knocking down USP22 up-regulate UBC to result in affecting lysosome pathway. (**C**) Knocking down USP22 up-regulates UBC to affect cell cycle pathway. (**D**) Knocking down USP22 could up-regulate UBC to affect the proteolysis pathway mediated by ubiquitin. Blue indicates down-regulated expression, while red indicates up-regulated expression.

**Figure 6:**
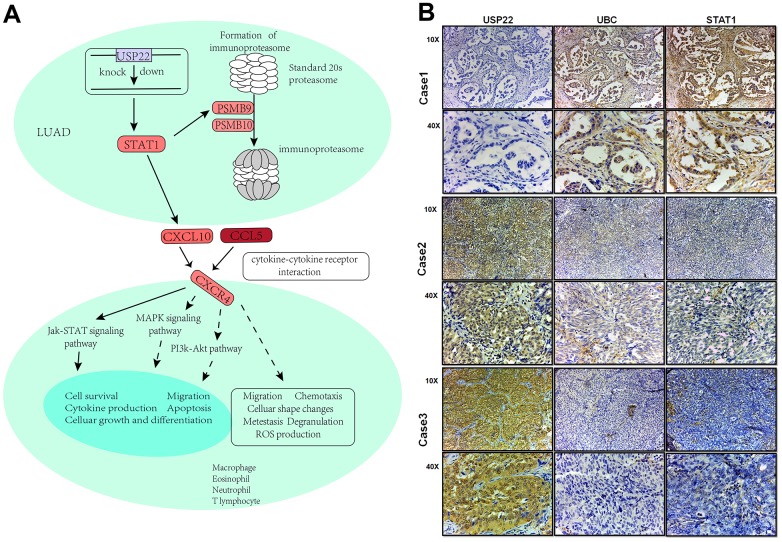
**Integrated landscape regulation of STAT1 by USP22-KD.** (**A**) Effect of knocking down USP22 on STAT1 signaling pathway. Blue indicates down-regulated expression, while red indicates up-regulated expression. (**B**) Detection of USP22, UBC and STAT1 by immunohistochemistry in LUAD.

USP22 mediated module function by regulating pivot genes in LUAD (Figure 5A). Knocking down USP22 can lead to up-regulation of UBC, which is involved in lysosomal, cell cycle, and ubiquitin-mediated proteolysis pathways (Figure 5B–5D). Up-regulation of UBC inhibited the occurrence of lysosomal autophagy, which may result in inhibition of normal cell apoptosis and promoting tumor cell proliferation. Our previous studies have confirmed that defects in USP22 can not only cause cell cycle arrest in G0/G1 phase, but also inhibit apoptosis and promote tumor cell proliferation [7]. After binding of E1 with UB, the UB complex is formed by E2 binding. Down-regulation of E2 can regulate enzyme, further resulting in inhibition of ubiquitination process and the production of the complex. Combining with E3, it can promote tumor proliferation, erosion and metastasis [9]. Moreover, STAT1 pathway could be activated by down-regulation of USP22 in LUAD H1975 cell line (Figure 6A), which can directly affect the formation of immune proteins and regulate JAK-STAT signaling pathway. As known to all, JAK1-STAT1-caspase pathway has been confirmed that it can induce apoptosis in non-small cell lung cancer (NSCLC) in previous studies. STAT1 is capable of maturation, proliferation and activation of NK cells. STAT1 signal triggering can also activate T cells to induce apoptosis of PD-L1-sensitive tumor cells. It suggests that STAT1 signal triggering may affect immune regulation, affecting the development of LUAD [10]. A huge impact arose on the STAT1 signaling pathway in knockdown of USP22, which will change the developmental process of tumor cells. According to the experiments, the expression of STAT1 and UBC in LUAD tissue were significantly lower than that in the control tissue (Figure 6B), while the high expression of UBC and STAT1 can inhibit the development of LUAD.

### Diagnostic and prognostic judgment of USP22-pivot gene

**Figure 7:**
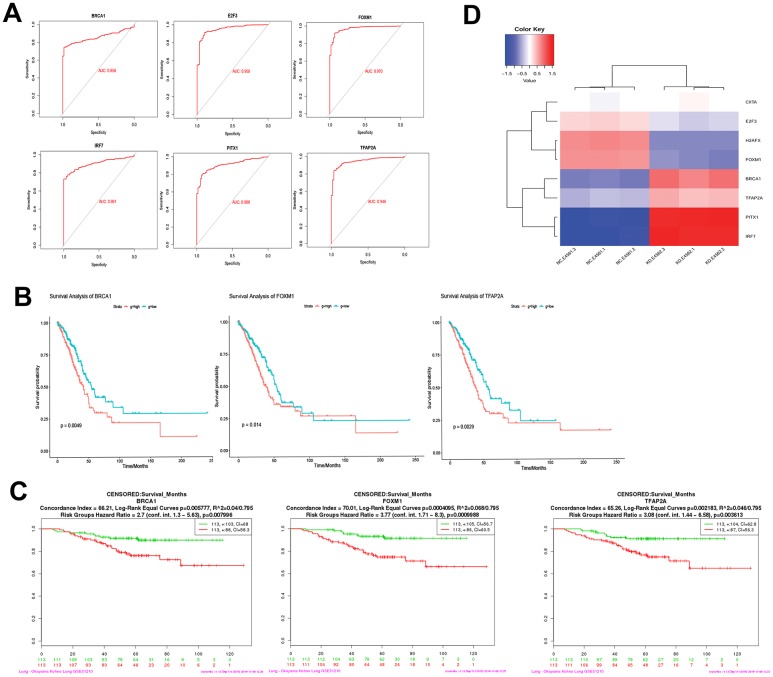
**Diagnostic ability and survival curve analysis of transcription factors.** (**A**) ROC curves of 6 genes prove that they have diagnostic ability for LUAD. (**B**) Survival curves of 3 genes most significantly correlated with LUAD. (**C**) The effect of 3 genes expression changes on prognosis was verified in GSE31210. (**D**) Transcription factors differential expression clustering thermogram, the differential expression of these eight pivot genes can be well distinguished USP22-KD H1975 cells and negative control.

ROC curve analysis suggested that E2F3, H2AFX, TFAP2A, PITX1, IRF7, and FOXM1 may be the potential biomarkers for LUAD (Figure 7A). BRCA1, FOXM1, and TFAP2A were significantly correlated with the prognosis of LUAD (Figure 7B). In addition, the effect of these gene expression changes on prognosis was also verified in GSE31210 (Figure 7C). Last but not least, the expression patterns of these mediator genes could accurately distinguish USP22-KD H1975 cells from NC (Figure 7D).

## DISCUSSION

USP22 protein was highly expressed in tumor tissues of LUAD patients, which was associated with a short survival time meanwhile [7, 11]. USP22 can promote the proliferation of tumors by deubiquitination-mediated proteolysis. The process of ubiquitination is closely related with various pathways involved in protein degradation, cell cycle regulation, apoptosis and DNA repair. There is important significance in deubiquitinating enzymes for the occurrence of tumors. Mutations and imbalances levels in proteins of tumor cells can unbalance tumor proteins and tumor suppressors in humans. When the balance is broken, it will stimulate the process of ubiquitination in the body, leading to a series of cellular responses. Subsequently, regulatory disorders result in the further development of the tumor. Overexpression of ubiquitinase can promote protein degradation. We confirmed that USP22 could induce the expression of UBC to inhibit the cell cycle, lysosomal degradation and ubiquitin-mediated proteolysis [1], which could further promote the occurrence and development of LUAD.

In recent years, immunotherapy has sprung up in the field of NSCLC. The current strategy is to interfere with immune checkpoints including cytotoxic T lymphocyte-associated antigen 4 (CTLA-4) or programmed cell death protein (PD-1). They are expressed in immune cells, which could prevent tissue damage by inhibiting the immune response. PD-1 immunotherapy has unique advantages in the treatment of non-small cell lung cancer (NSCLC) [12]. Although immunotherapy has achieved amazing achievement in the treatment of various cancers, such as NSCLC and malignant melanoma, the potential mechanisms still remained poorly understood. The immunoproteasome is an important part of the immune system [13, 14]. The deletion of immunoproteasome in NSCLC may be associated with greater invasiveness. Researchers believe that the imbalance of signal transduction STAT1 and transcription activators STAT3 can lead to that [15–17], suggesting that recovery the expression of immunoproteasome may be a new way to improve T cell mediated to immunotherapy. At the same time, immune functions except for antigen presentation can also be regulated by immunoproteasome, including the production of cytokines through NF-κB pathway, the expansion of T cells and the differentiation of T helper cells, etc. [13]. What more excited is that USP22 can inhibit the antitumor immunity, efficacy of PDL1 targeted immunotherapy by inhibiting the deubiquitinase of PDL1 (CD274) [18], which is consistent with our results. We confirmed that USP22 could participate in the interaction between tumor cells and immune cells. Through regulation of STAT1 mediator genes, it can participate not only in non-specific immune process but also in specific immune cell activities. Cell experiment shows that, compared to knocking down USP22, a stronger cell proliferation ability in LUAD parental cell lines. At the same time, we confirmed that knocking down USP22 can lead to activation of STAT1 signaling. This process can regulate PSMB9 and PSMB10 to directly promote the formation of immunoproteasome, which may activate the JAK-STAT signaling pathway through CXCL10 and CCL5. Activated STAT1 can promote the maturation, proliferation and activation of NK cells. Activation of NK cells can recognize and eliminate cancer cells or virus-infected cells [19]. More and more studies show that STAT1 mediated IFN signal is a key step in monitoring the immune system to recognize and eradicate emerging tumor [20, 21]. Up regulation of IFN mediated by STAT1 signal can be used in immunomodulating to avoid the risk of tumor occurrence, such as immune escape [22]. At the same time, STAT1 signal triggering can activate T cells and induce apoptosis of PD1-sensitive tumor cells. It is known that over-induction of PD1 can lead to depletion of T cell and immune escape of tumor cells [12].

Knocking down the expression of USP22 can activate STAT1 signaling pathway, inhibiting the depletion of T cells, promoting the production of immune cells and the death of other non-specific immune cells. It suggests that USP22 may be a potential drug target as a potential novel immunotherapy target.

Expect for the recognition of these pathway mechanisms, we confirmed that E2F3, H2AFX, TFAP2A, PITX1, IRF7 and FOXM mediator genes can be used as molecular markers for the diagnosis of LUAD. Among them, BRCA1, FOXM1 and TFAP2A all have potential prognostic judgment ability for LUAD patients. Especially, E2F3 was confirmed that the expression increased significantly in lung cancer tissues [23]. Furthermore, TFAP2A was confirmed to inhibit the development and progression of lung cancer, as well as the disordered pathway of FENDRR (FOXF1 adjacent non-coding development regulatory RNA). A series of studies corroborate that overexpression of FOXM1 and PTIX1 can promote the proliferation of lung cancer cells [24, 25]. Study shows that activated IRF7 can promoted NSCLC cell proliferation, invasion and migration while inhibiting cell apoptosis [26], while in this study, we found out that it could be used as a diagnostic marker. H2AFX histone mutation plays a key role in DNA repair, which may affect cancer susceptibility [27]. USP22 induces the occurrence and development of LUAD by promoting the expression of H2AFX histone.

In this study, we speculated that USP22 might be involved in a variety of ways to promote the development of LUAD, including ubiquitination and immunosuppression. The results still need to be verified by subsequent experiments. Due to it is based on the results of bioinformatics analysis and prediction, which can be used as a candidate resource and early basis for scientific team to carry out experimental exploration.

In conclusion, we constructed a USP22-pivot gene-module-pathway global regulation network. Based on the network, we propose that USP22 could drive multiple pivot genes to regulate ubiquitination and immunosuppression, further promoting the development of LUAD.

## MATERIALS AND METHODS

### Tissue collection and immunohistochemical analysis of LUAD patients

We selected the wax blocks from the surgically resected tissues of 110 patients with lung adenocarcinoma as experimental subjects from January 2006 to December 2007. At the same time, the clinic pathological data of the study cases was collected (Table 1). None of the patients received preoperative chemotherapy or radiotherapy. Primary cancers were assessed according to the American Cancer Joint Commission's 7^th^ edition staging system. The Hospital's Ethics Committee approved the study.

Human LUAD cell line H1975 was preserved in the laboratory of Institute of Oncology, Harbin Medical University. Cell lines were cultured in RPMI-1640 medium containing 5% FBS and cultured at 37 degree C and 5% CO_2_. The short-tandem repeat (STR) method was applied to identify the cell lines regularly.

We collected pathological information from Harbin Medical University Cancer Hospital. There were 110 patients with lung adenocarcinoma, who underwent surgical treatment from January 2006 to January 2007, included in the study. The specimens were fixed with formaldehyde and embedded in paraffin. Immunohistochemistry was applied in the lung tissues of patients that were diagnosed as LUAD. The study has been approved by the Ethics Committee of Harbin Medical University Cancer Hospital. Written informed consent was obtained from all patients.

### Immunohistochemistry

Immunohistochemistry (IHC) was performed to detect the expression of USP22, UBC and STAT1 in LUAD. IHC method and criteria for judging results were referred to literature [7, 28]. Under high power microscopy (10 x 40), the percentage count of positive cells was determined by enumerating 500 cells in five random regions, the staining intensity and percentage of positive cells were calculated. Immunohistochemical staining was scored according to the following criteria: “-” indicated no positive staining cells, “+” indicated 1-20% positive cells, “++” indicated 20-50% positive cells, “+++” indicated 50-100% positive cells. IHC expression of evaluating proteins: “- ~ +” was defined as low expression; “++ ~ +++” was defined as high expression. All IHC stained pathological films were confirmed by at least two pathologists in a double-blind manner. The antibodies were (ABclonal, A6173, 1:100) for USP22, (ABclonal, A3207, 1:100) for UBC, and (Proteintech, 10144-2-AP, 1:40) for STAT1.

### Lentivirus interference

H1975 cells were infected with lentiviruses expressing either scrambled shRNA (negative control, NC) or specific shRNA for knocking down USP22 (USP22-KD). Lentiviral interference technology knocked down the expression of USP22 in H1975 cells. According to the design principle of RNAi sequence, multiple RNAi target sequences were designed, in which the best kinetic parameter target was selected to enter the subsequent experiments [8]. H1975 cells were cultured in excellent growing conditions. The experimental conditions were designed, according to the preliminary results of lentivirus infection, which was a puromycin-labeled lentivirus infection. After 48-72 hours of infection, antibiotics were screened for 48 hours. Quantitative Real-time PCR was used to verify the knockdown efficiency of USP22. Knockout efficiency of USP22 genes reached 68.2% (Supplementary Table 1). In H1975 cells, USP22-KD group (n = 3) and NC group (n = 3) were set up for subsequent microarray sequencing. There are three sequence repeats.

### Microarray processing

Total RNA was extracted from these cells by using Trizol reagents. RNA quantity and quality were assessed by using a NanoDrop 2000. According to the manufacturer's instructions and previous studies, microarray processing was performed to determine gene expression profiling using the Affymetrix human GeneChip Primeview (ThermoFisher Scientific, Catalog number: 901837) [8, 29]. Raw array data were produced by scanning with the Gene Chip Scanner 3000.

### Bioinformatics analysis

### Gene set enrichment analysis (GSEA)

The processed gene expression profiles were applied to performing GSEA [30], to explore the Kyoto Encyclopedia of Genes and Genomes (KEGG) pathways that were related to USP22-KD H1975 cells. GSEA was performed by using GSEA JAVA software (http://software.broadinstitute.org/gsea/index.jsp). Gene sets c2.cp.kegg.v6.2.symbols.gmt [31] was the reference gene sets. false discovery rate (FDR) < 0.25 was considered significant.

### Screening differentially expressed genes between USP22-KD H1975 cells and NC H1975 cells

The processed gene expression profiles were used to screen differentially expressed genes (DEGs) between USP22-KD H1975 cell lines (n = 3) and control parental H1975 cell lines (n = 3). DEGs screening was performed by using limma package [32] in R, with the criteria of P adjusted FDR< 0.01 and |log2 fold change| > 1.

### Modular analysis of protein-protein interaction (PPI) networks and functional enrichment analyses

According to STRING database [33] (https://string-db.org/), we constructed PPI networks of DEGs. Cytoscape software [34] was used to network visualization. The ClusterONE [35] plugin in Cytoscape software was applied to perform a modular analysis of the PPI network (Minimum = 30). The DEGs were organized into multiple functional modules. The ClusterProfiler package [36] in R was used to performed functional enrichment analyses for these functional modules. Functional enrichment analysis included biological process (BP) and KEGG pathway in the present study. P adjusted by FDR < 0.01 was considered as significant.

### Correlation analysis and hypergeometric test

Based on the STRING database, we extracted DEGs, which interact with USP22. Their interactions with USP22 were validated with Pearson correlation in the present study. P < 0.05 was considered significant. We hypothesized that these genes might be the pivots of the USP22 regulatory function module. To verify the hypothesis, hypergeometric test was performed by igraph package (https://igraph.org/r/) in R, in which P value of < 0.05 was considered significant. Subsequently, we constructed a USP22-pivot-pathway global transcriptional regulation network.

### Survival analysis and receiver operating characteristic (ROC) curve analysis

The median expression value for a pivot gene was considered as the cutoff value, to divide patients into high-expression and low-expression groups. In order to explore the correlation between the expression level of pivot gene and the overall survival time, we used the log rank method to carry out Kaplan Meier survival analysis. The survival package (https://CRAN.R-project.org/package=survival) in R was used to perform the survival analysis. Moreover, survminer package (https://CRAN.R-project.org/package=survminer) in R was used to visualize the survival curve, in which P < 0.01 was considered to be significant. ROC curve analysis was carried out by using pROC package [37], to assess diagnostic value of the gene. Furthermore, we verified the identified genes in SurvExpress (http://bioinformatica.mty.itesm.mx) web tools, using GSE31210 from Gene Expression Omnibus (https://www.ncbi.nlm.nih.gov/geo/).

### Ethics statement

All institutional and national guidelines for the care and use of laboratory animals were followed. This article does not contain any studies with human subjects performed by any of the author

## Supplementary Material

Supplementary Figure 1

Supplementary Table 1
