# Supplementary material for: USP22 promotes development of lung adenocarcinoma through ubiquitination and immunosuppression
Source: Aging (Albany NY). 2020 Apr 15;12(8):6990–7005. doi: 10.18632/aging.103056 (PMC7202522; doi:10.18632/aging.103056)
Supplement: Supplementary Figure 1 [file aging-12-103056-s002..pdf]

SUPPLEMENTARY FIGURE

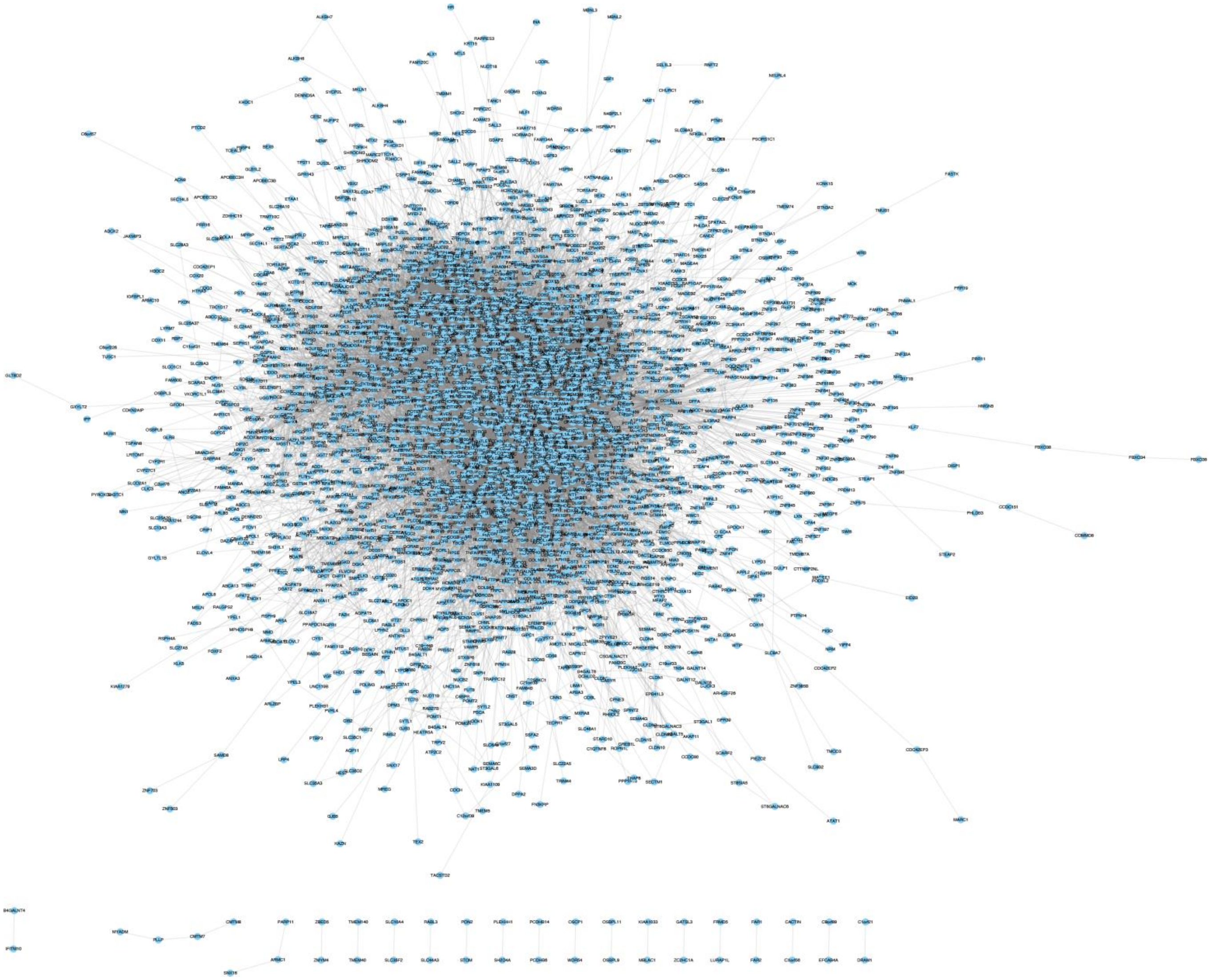

Supplementary Figure 1. Protein-protein interaction network of differentially expressed genes between USP22-KD and negative control H1975 cells.
