# Supplementary material for: USP22 promotes development of lung adenocarcinoma through ubiquitination and immunosuppression
Source: Aging (Albany NY). 2020 Apr 15;12(8):6990–7005. doi: 10.18632/aging.103056 (PMC7202522; doi:10.18632/aging.103056)
Supplement: Supplementary Table 1 [file aging-12-103056-s001..pdf]

SUPPLEMENTARY TABLE

Supplementary Table 1. Knock down efficiency data analysis of USP22.

Sample No.	Group marking	Data analysis								Hole difference		
		GAPDH	USP22	ΔCt	-ΔΔCt	2-ΔΔCt	Expression abundance	STDEV.	p value	Reference gene	Target gene	Knock down efficiency
E4561	NC	15.09	28.17	13.08	0.150	1.110	1.009	0.161		0.170	0.330	
		15.16	28.26	13.1	0.130	1.094						
		14.99	28.5	13.51	-0.280	0.824						
E4562	KD	13.91	28.73	14.82	-1.590	0.332	0.318	0.020	0.016	0.020	0.160	0.682
		13.89	28.73	14.84	-1.610	0.328						
		13.9	28.89	14.99	-1.760	0.295						
